# Na_4_PMo_11_VO_40_-catalyzed one-pot oxidative esterification of benzaldehyde with hydrogen peroxide†

**DOI:** 10.1039/d1ra06718d

**Published:** 2021-10-28

**Authors:** Castelo Bandane Vilanculo, Márcio José da Silva

**Affiliations:** Chemistry Department, Pedagogic University of Mozambique, FCNM, Campus of Lhanguene Av. De Moçambique, Km 1 Maputo 4040 Mozambique castelovilanculo@gmail.com +258 825573337; Chemistry Department, Federal University of Viçosa Minas Gerais State 36590-000 Brazil

## Abstract

The activity of the sodium salts of vanadium-doped phosphomolybdic acid was assessed in the oxidative esterification reaction of benzaldehyde with hydrogen peroxide in alkyl alcohol solutions. The effect of main reaction parameters, such as temperature, catalyst load, vanadium doping level, and reactant stoichiometry, on the conversion and reaction selectivity was investigated. Among the tested heteropoly salts, Na_4_PMo_11_VO_40_ was the most active and selective catalyst, achieving almost complete conversion of benzaldehyde and high ester selectivity regardless of the alcohol investigated. The efficiency of the catalyst was correlated with its vanadium content. The size of the carbon chain of alcohol and the steric hindrance on the hydroxyl group played a key role in the reaction selectivity. While methyl and ethyl alcohols selectively provided the ester as the main product (*ca.* 90–95%) and benzoic acid as a subproduct, the other alcohols also afforded acetal, a condensation product, and benzaldehyde peroxide, an oxidation reaction intermediate, as secondary products. The use of an inexpensive, environmentally benign, and atom-efficient oxidant, mild conditions, and short reaction times were the positive aspects of this one-pot process.

## Abstract 2

The activity of the sodium salts of vanadium-doped phosphomolybdic acid was assessed in the oxidative esterification reaction of benzaldehyde with hydrogen peroxide in alkyl alcohol solutions.

## Graphical abstract

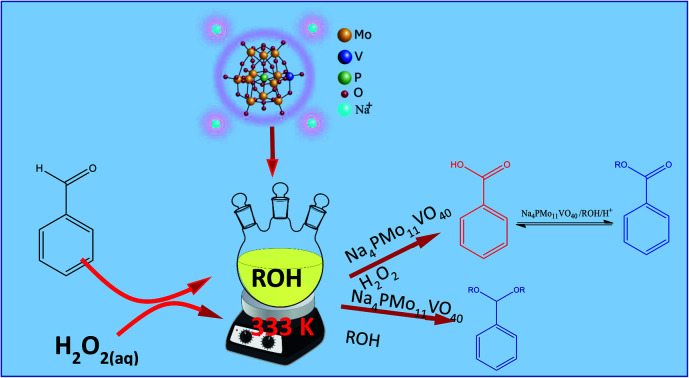

## 1. Introduction

The synthesis of aromatic esters has gained attention due to the wide utility of these compounds as raw materials in the industrial production of resins, perfumes, cosmetics, fibers, plasticizers, and dyes.^1^ However, the traditional routes of ester production involve hazardous and environmentally unfriendly reagents, which leads to large generation of stoichiometric quantities of residues and effluents.^2^

To circumvent these drawbacks, alternative routes to the traditional stoichiometric oxidation processes, such as the direct transformation in one-pot reactions of aldehydes into esters, have been developed.^3^ Indeed, the oxidative esterification of aldehydes has been raised as a sustainable and efficient alternative to classical synthesis.^4,5^ Several Lewis acid metal-catalyzed reactions using tertbutyl peroxide or hydrogen peroxide as an oxidant have been described.^6–9^

In this sense, hydrogen peroxide is an easy-handling liquid reactant that is inexpensive, atom-efficient, and non-flammable; it is also a green oxidant that generates only water as a by-product.^10,11^ The choice of an adequate solvent avoids the use of a phase transfer agent as well as the addition of pH controllers.^12–14^ Nonetheless, hydrogen peroxide requires an activation step, which is generally performed by a metal catalyst such as an oxide, salt, or organometallic compound.^15–18^

Solid-supported catalysts have been demonstrated to be active in the oxidation of aldehydes with hydrogen peroxide.^19–23^ Thakur *et al.* investigated oxidation with hydrogen peroxide of aromatic and aliphatic aldehydes to methyl esters over a VO(acac)_2_/TiO_2_ catalyst.^24^ In this sense, vanadium catalysts have been highlighted as effective catalysts in several oxidation reactions, mainly when used in Keggin heteropoly compounds. Keggin heteropolyacids (HPAs) are polyoxometalates that are widely used as catalysts due to their acidic and redox properties; thus, they are potentially active in oxidative esterification reactions.^25,26^ They have been widely used as catalysts in oxidation reactions with hydrogen peroxide.^27–30^

Keggin HPAs are well-defined metal–oxygen clusters in which oxygen atoms link tungsten or molybdenum atoms, resulting in octahedral units that are tetrahedrally arranged around a heteroatom (*i.e.*, phosphorus or silicon atom).^31,32^ Because Keggin HPAs are soluble in polar solvents, they have been used as solid-supported catalysts.^33,34^ Alternatively, Keggin HPAs can be converted to solid salts, exchanging their protons by larger radium cations as cesium or potassium.^35,36^

Two other interesting approaches may improve the performance of Keggin HPAs in catalytic oxidation reactions: the removal of an MO unit (*i.e.*, M = W or Mo), generating lacunar catalysts,^37,38^ and the exchange of an addenda atom (*i.e.*, tungsten or molybdenum) by vanadium atom.^39,40^ Lacunar Keggin HPA catalysts were successfully used in the oxidation of aldehydes with hydrogen peroxide.^41,42^

On the other hand, the simple exchange of Mo or W by V atoms in the primary structure of a heteropolyanion can accelerate the steps of oxidation–reduction, enhancing the activity and selectivity of oxidation reactions.^43–45^ Particularly, molybdenum-based HPAs have been found to be better catalysts for oxidation reactions than their tungsten counterparts.^46,47^ Moreover, vanadium-doped phosphomolybdic catalysts have been generally used as acids, an aspect that can compromise the selectivity of oxidation reactions.

In this work, for the first time as far as we know, sodium phosphomolybdate salts were doped with different vanadium loads and used as catalysts in oxidative esterification reactions of benzaldehyde with hydrogen peroxide in alcoholic solutions. The focus was to assess the impacts of the vanadium load on the conversion and selectivity of the reactions. To accomplish this, sodium salts of phosphomolybdic acid, containing Keggin-type heteropolyanions with the general formula PMo_12−*n*_V_*n*_O_40_^(3+*n*)−^ (*n* = 0, 1, 2, or 3), were synthesized and evaluated. The effects of the main reaction parameters of the reaction, such as the oxidant load, type, and concentration of the metal catalyst, temperature, and nature of the alcohol, were investigated.

## 2. Experimental

### 2.1. Materials and methods

All chemicals were purchased from commercial sources. Benzaldehyde and alkyl alcohols (*i.e.*, methyl, ethyl, propyl, butyl, *sec*-propyl, *sec*-butyl) were all obtained from Sigma-Aldrich (99 wt%). Hydrogen peroxide was obtained from Moderna (34 wt%). Hydrated heteropolyacid H_3_PMo_12_O_40_ (99 wt%) was acquired from Sigma-Aldrich. V_2_O_5_ (99.6 wt%), MoO_3_ (99.5 wt%), H_3_PO_4_ (85 wt%), NaVO_3_ (98 wt%), Na_2_MoO_4_ (≥98 wt%), and CH_3_CN (99 wt%) were also purchased from Sigma-Aldrich. Aqueous hydrogen peroxide (35 wt%) was acquired from Alphatec, and H_2_SO_4_ (95–98 wt%) was obtained from Dinâmica.

### 2.2. Synthesis of the Na_4_PMo_11_VO_40_, Na_5_PMo_10_V_2_O_40_, and Na_6_PMo_9_V_3_O_40_ catalysts

**Scheme 1:**
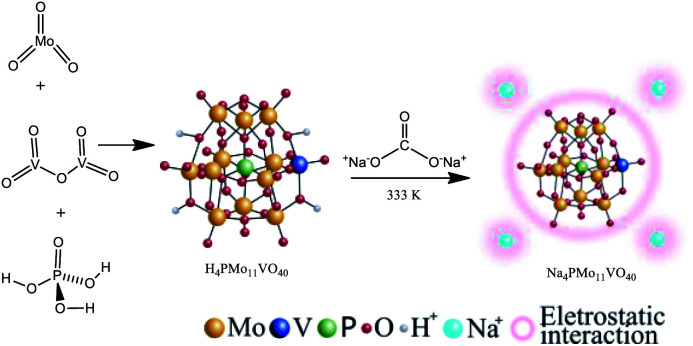
Route of synthesis of Na_4_PMo_11_VO_40_.

The Na_4_PMo_11_VO_40_ salt was synthesized according to the literature as depicted in Scheme 1.^48^

Typically, stoichiometric amounts of MoO_3_ and V_2_O_5_ were dissolved in deionized water and heated to the boiling point. Then, phosphoric acid was added, and the resulting mixture was refluxed for 6 h. Cooling to room temperature afforded a clean solution. Evaporation of the solvent resulted in the solid acid (*i.e.*, H_4_PMo_11_VO_40_), which was recrystallized. This solid was dissolved in aqueous, and an aqueous solution of sodium carbonate was added; the mixture was stirred and heated at 333 K for 3 hours. Finally, evaporation of the solvent led to the Na_4_PMo_11_VO_40_ salt, which was recrystallized from water and then dried at 373 K/5 h.

### 2.3. Synthesis of the Na_5_PMo_10_V_2_O_40_ and Na_6_PMo_10_V_3_O_40_ salts

**Scheme 2:**
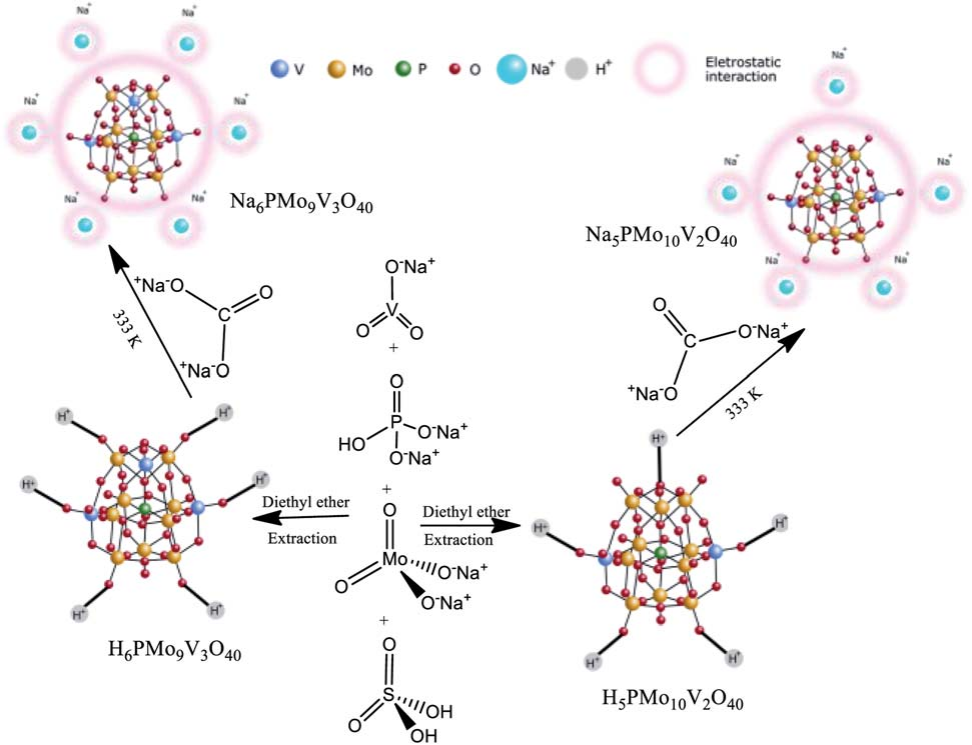
Route of synthesis of Na_5_PMo_11_V_2_O_40_ and Na_6_PMo_11_V_3_O_40_.

These catalysts were synthesized according to the original and modified procedures.^49,50^ An aqueous solution of sodium diphosphate was added to a hot aqueous solution of sodium *meta*-vanadate in an adequate stoichiometric ratio (Scheme 2).

After the mixture was cooled to room temperature, concentrated sulfuric acid (*ca.* 5 mL) was slowly added, and the solution developed a red color. Subsequently, a solution of sodium molybdate was added under vigorous stirring. Sulfuric acid (*ca.* 85 mL) was slowly added, and the solution was cooled to room temperature. The etherate extract was vapored under airflow, affording the solid acid H_5_PMo_10_V_2_O_40_. The resulting solid was dried at 343 K and later dried at 373 K/5 h. The solid acid was solved in water, and a Na_2_CO_3_ solution was added, mixed, and heated at 333 K for 3 hours. Evaporation of the water afforded a Na_5_PMo_10_V_2_O_40_ salt, which after recrystallization was dried at 373 K/5 h.

A similar procedure was used to synthesize H_6_PMo_9_V_3_O_40_ and the Na_6_PMo_9_V_3_O_40_ salt, except by taking the required amounts of sodium metavanadate and sodium molybdate. In this case, the resulting solution became cherry red.

### 2.4. Catalyst characterization

Infrared spectroscopy analyses were recorded on a Varian 660-IR spectrometer at 400 to 1300 cm^−1^ wavenumbers, which is the fingerprint region of the typical absorption bands of Keggin anions. UV-visible spectroscopy analyses were obtained in a AJX-6100 PC double beam Micronal spectrometer fitted with tungsten and deuterium lamps. The spectra were recorded from CH_3_CN solutions with concentrations of 0.002 mol L^−1^, the concentration used in the catalytic reactions.

Powder X-ray diffraction patterns of the vanadium heteropoly salts were obtained using an X-ray diffraction system (model D8-Discover, Bruker) using Ni-filtered Cu-kα radiation (*λ* = 1.5418 Å), working at 40 kV and 40 mA, with a counting time of 1.0 s in an angle (2*θ*) range from 5 to 80 degrees.

The porosimetry of the catalysts was analyzed by N_2_ adsorption/desorption using a NOVA 1200e High Speed Automated Surface Area and Pore Size Analyzer (Quantachrome Instruments). The samples were previously degassed for 1 h. The surface areas of the salts were calculated by applying the Brunauer–Emmett–Teller equation (BET) to the desorption/adsorption isotherms. Thin sections of salts were selected to characterize their surfaces, which were metalized with carbon and analyzed through scanning electron microscopy (SEM) and energy-dispersive X-ray spectroscopy (EDS) using a JEOL JSM 6010LA SEM.

The catalyst acidity was estimated by potentiometric titration, as described by Pizzio *et al.*^51^ The electrode potential variation was measured with a potentiometer (*i.e.*, Bel, model W3B). Typically, 50 mg of vanadium salts were dissolved in CH_3_CN and then titrated with *n*-butylamine toluene solution (*ca.* 0.05 mol L^−1^).

### 2.5. Catalytic runs

Catalytic tests were carried out in a glass reactor (*ca.* 25 mL), fitted with a reflux condenser and sampling septum, in a glycerine bath. Typically, benzaldehyde (*ca*. 2.75 mmol) and the vanadium heteropoly salt catalyst (*ca.* 1.77 mol%) were dissolved in C_2_H_5_OH (*ca.* 10 mL solution) at reaction temperature (*ca.* 333 K). The solution was magnetically stirred, and aqueous H_2_O_2_ solution (*ca.* 8.25 mmol) was slowly added, starting the reaction.

The reactions were followed by GC analysis of regularly collected samples (GC 2010 Shimadzu, capillary column, FID). The reaction products were identified by GC-MS analysis (GC-MS 2010 *ultra mass*, *i.e.*, 70 eV) and co-injection in GC equipment with analytical patterns (*i.e.*, benzoic acid), or by comparison with authentic samples that were previously synthesized (*i.e.*, acetal and esters). The mass balance of the reaction was checked by comparing the GC peak area of the substrate consumed with the sum of the corrected GC peak area of the main products.

The reaction conversions were calculated through eqn (1), comparing the GC peak area of benzaldehyde in each reaction (*A*_i_) with the initial area (*A*_0_).

1 % conversion = (*A*_0_ − *A*_i_)/*A*_0_ × 100

The selectivity was calculated from eqn (2), where the corrected area of the GC peak of each product (*A*_p_) was compared to the initial area of the GC peak of benzaldehyde (*A*_0_) – eqn (2)

2 % selectivity = (*A*_p_/*A*_0_) *×* 100

The selectivity of benzaldehyde peroxide, which is an undetected product by GC analysis, was calculated using eqn (3).

3 % benzaldehyde peroxide = (consumed GC peak area of benzaldehyde − ΣGC peak corrected area of products)/consumed GC peak area of substrate × 100

## 3. Results and discussion

### 3.1. Catalyst characterization

**Fig. 1:**
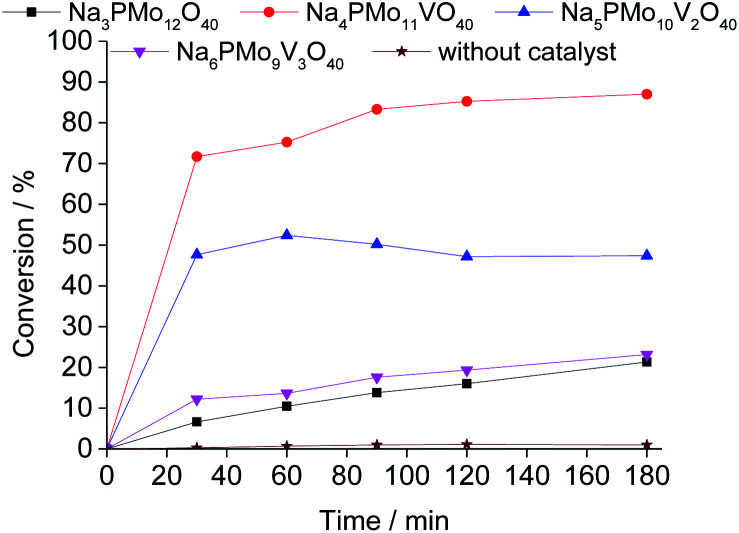
Kinetic curves of oxidative esterification reactions of benzaldehyde with H_2_O_2_ in C_2_H_5_OH solutions in the presence of undoped and banadium-doped sodium phosphomolybdate catalysts. Reaction conditions: benzaldehyde (2.75 mmol), H_2_O_2_ (8.25 mmol), catalyst (1.77 mol%), toluene (0.1 mL), C_2_H_5_OH (10.0 mL), 333 K.

**Fig. 2:**
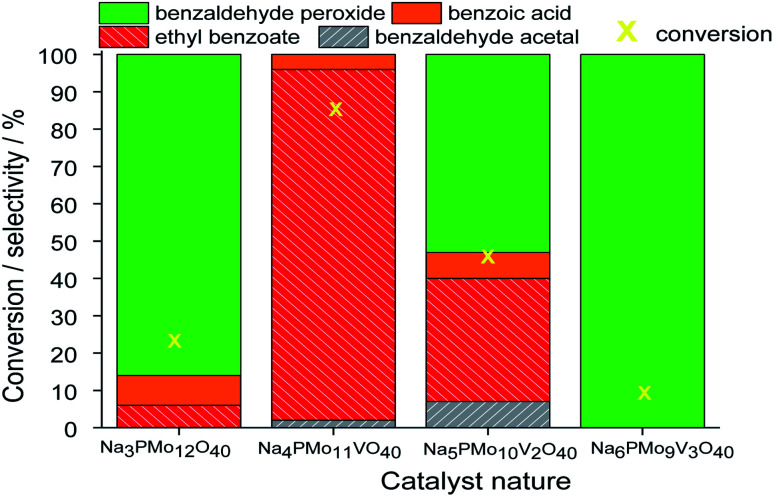
Effect of the catalyst nature on the conversion and selectivity of oxidative esterification reactions of benzaldehyde with H_2_O_2_ in C_2_H_5_OH in the presence of undoped and vanadium-doped sodium phosphomolybdate catalysts. Reaction conditions: benzaldehyde (2.75 mmol), H_2_O_2_ (8.25 mmol), catalyst (1.77 mol%), toluene (0.1 mL), C_2_H_5_OH (10.0 mL), 333 K.

**Fig. 3:**
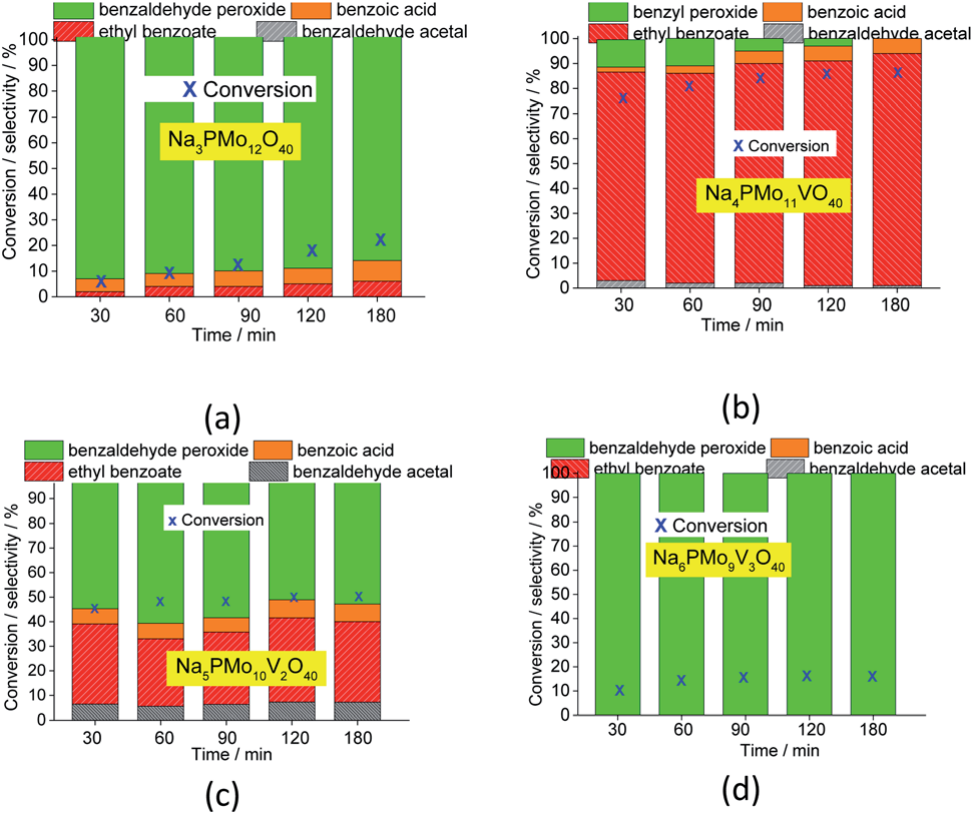
Variations of the conversion and selectivity of oxidative esterification reactions of benzaldehyde with H_2_O_2_ in C_2_H_5_OH solutions in the presence of pristine (a) and vanadium-doped (b–d) sodium phosphomolybdate catalysts. Reaction conditions: benzaldehyde (2.75 mmol), H_2_O_2_ (8.25 mmol), catalyst (1.77 mol%), toluene (0.1 mL), C_2_H_5_OH (10.0 mL), 333 K.

The characterization of the vanadium-doped sodium phosphomolybdate catalysts was previously discussed in other work, where they were used in the epoxidation of terpene alcohols with hydrogen peroxide.^52^ Notwithstanding, all the important data obtained in the characterization (*i.e.*, infrared spectra, powder XRD patterns, EDS analyses, and measurements of the strength of the acidic sites) are shown in the supplemental material (Fig. 1SM–3SM†). The most important characterization data and the respective assumptions are summarized as follows:

• The integrity of the primary structures of the vanadium-doped heteropolyanions was confirmed by infrared spectroscopy analysis (Fig. 1SM†). The fingerprint region of the infrared spectra presents the typical absorption bands of a Keggin anion.^53^ A comparison of the infrared spectra of the vanadium salts and undoped sodium phosphomolybdate salt clearly showed that the primary structure of the catalyst (*i.e.*, Keggin heteropolyanion) remained almost untouched after the synthesis of the salts (Fig. 1SM†). A more detailed characterization and better discussion of the infrared spectra of these catalysts was previously presented when they were used in oxidation reactions of terpenic alcohols.^52^

• The analysis of the powder XRD patterns allows us to verify if any changes occurred in the secondary structure of Keggin HPAs when their protons were exchanged by sodium cations and when vanadium ions were introduced into the heteropolyanion. X-Ray diffractograms of the sodium phosphomolybdate salts evidenced that vanadium doping increased the level of crystallinity, preserving the main diffraction peaks between the 5 and 40° 2*θ* angles. This suggests that both the primary (*i.e.*, Keggin anion) and secondary structures were preserved. New diffraction signals were noticed in the low angle region (*ca.* 2*θ* 10°) of all the XRD diffractograms of the salts. Moreover, new diffraction peaks at 2*θ* angles greater than 40° (*ca.* 47° and 50° angles) appeared in the diffractogram of the monosubstituted salt (Fig. 2SM†). These changes are attributed to the difference between the ionic radius of the hydrate protons (*i.e.*, H_3_O^+^, H_2_O_5_^+^) and Na^+^ ions, which may affect the packaging of the heteropolyanions on the secondary structure as well as the different hydration levels of the salts.^54^ A comparison of the XRD patterns of the sodium salts with their respective acids allow us to conclude that the sodium salts presented a body-centered cubic structure, which remained almost intact after the inclusion of one vanadium atom.^55,56^

• The strength of the acidic sites of the phosphomolybdic catalysts was estimated by measuring the initial electrode potential (*i.e.*, *E*_i_) of their acetonitrile solutions (Fig. 3SM†). While the phosphomolybdic acid displayed a value of *E*_i_ = 680 mV,^52^ its unsubstituted and vanadium-monosubstituted sodium salts presented *E*_i_ values equal to 400 and 370 mV, respectively. However, an increase in vanadium load drastically reduced the acidity strength of the sodium phosphomolybdate salts; the values of *E*_i_ measured in the solutions of Na_5_PMo_10_V_2_O_40_ and Na_6_PMo_9_V_3_O_40_ salts were equal to 65 and −100 mV, respectively (Fig. 3SM†). According to the literature, the two first salts have very strong acidic sites (*E*_i_ > 100 mV) and Na_5_PMo_10_V_2_O_40_ has strong acidic sites (0 < *E*_i_ < 100 mV), while the Na_6_PMo_9_V_3_O_40_ salt has weak acidic sites (−100 < *E*_i_ < 0 mV).^51^

• The MEV images obtained from the vanadium-doped phosphomolybdate sodium salts revealed that the particles of Na_4_PMo_11_VO_40_ are smaller than those of the undoped Na_3_PMo_12_O_40_. Therefore, the vanadium doping increased their surface area, as demonstrated by BET analysis.^52^ In the elemental analysis, the percentual elemental compositions of the sodium phosphomolybdate salts were confirmed by EDS analysis.^52^

• The hydration levels of the vanadium-doped phosphomolybdate sodium salts were determined by TG/DTG analyses.^52,57^ It was verified that upon increasing the number of vanadium atoms doped into the heteropolyanion, the number of water molecules increased (*ca.* 7, 10, and 13 water moles when 1, 2, or 3 vanadium atoms were doped into the anion, respectively). Some of the changes observed in the XRD patterns of the sodium salts (Fig. 2SM†) can be assigned to the distinct hydration levels.^52^ Finally, the DSC analyses of the phosphomolybdate salts also confirmed that the vanadium doping increased the thermal stability.^52^

### 3.2. Catalytic tests

#### 3.2.1. Screening of the vanadium-doped sodium phosphomolybdate salt catalysts

Herein, our initial aim was to verify the vanadium doping level required to achieve the highest conversion and selectivity in the oxidative esterification of benzaldehyde with hydrogen peroxide. Initially, the reactions were carried out in ethyl alcohol solution using a catalyst load of 1.77 mol% and a 3 : 1 molar ratio of oxidant to substrate.

Comparing the performance of the sodium phosphomolybdate catalysts with and without vanadium, we can realize that the doping triggered a noticeable improvement in the activity of the heteropoly catalyst. However, when the content of vanadium was increased from V1 to V2 or V3, a decrease in the conversion of the reactions was noticed (Fig. 1).

**Scheme 3:**
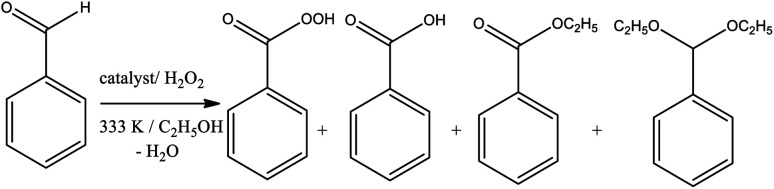
Products of the sodium phosphomolybdate-catalyzed oxidative esterification of benzaldehyde with hydrogen peroxide in ethyl alcohol solutions.

In terms of selectivity, it was clear that the Na_4_PMo_11_VO_40_ salt was the most efficient catalyst (Fig. 2). In general, 4 products were formed in all the reactions (Scheme 3). Benzaldehyde peroxide and benzoic acid were oxidation products and ethyl benzoate was the product of oxidation, followed by esterification; also, benzaldehyde acetal was formed through the condensation reaction of benzaldehyde and ethyl alcohol.

While the reaction rate was almost unaffected, the vanadium content had a remarkable impact on the product distribution (Fig. 3). Without vanadium, the reaction does not provide oxidation products in a significant amount. Conversely, the Na_4_PMo_11_VO_40_-catalyzed reaction achieves the maximum conversion (*ca.* 90%) and highest ester selectivity within the first reaction hour (Fig. 3). From this vanadium content, as the load increased, the conversion and the ester selectivity decreased. Noticeably, the efficiency of Na_6_PMo_9_V_3_O_40_ was lower than that of Na_3_PMo_12_O_40_. This effect deserves to be investigated in future work.

In all the other reactions, in addition to the lower conversions, benzaldehyde peroxide, which is an intermediate of the oxidation reaction, was the main product. Conversely, in the Na_4_PMo_11_VO_40_-catalyzed reaction, methyl benzoate, a product of one-pot oxidative esterification, was more selectively formed. It is probably obtained through consecutive steps of oxidation to benzoic acid and esterification with methyl alcohol reactions. The same effect was observed when benzaldehyde was oxidatively esterified over TiO_2_-supported vanadium-doped cesium phosphomolybdate (*i.e.*, Cs_3+*n*_PMo_12−*n*_V_*n*_O_40_/TiO_2_, *n* = 0–3) catalysts.^58^ Indeed, despite the different oxidants used by those authors (*i.e.*, molecular oxygen), they verified that while the monosubstituted supported catalyst (*i.e.*, Cs_4_PMo_11_VO_40_/TiO_2_) achieved the highest conversion, a greater vanadium content led to a lower conversion.

Fig. 3 presents the reaction selectivity variation with time for the 4 sodium phosphomolybdate catalysts. It is possible to observe that among the vanadium salts, only Na_4_PMo_11_V_1_O_40_ was an efficient catalyst. Indeed, when vanadium was included in the phosphomolybdic anion, there was a significant gain in catalytic performance, either in conversion or oxidation selectivity (Fig. 3a and b). However, an increase in vanadium doping drastically reduced the activity and selectivity of the catalyst.

Recently, we have found that an increase in vanadium doping led to a decline in conversion reached in oxidation reactions of terpenic alcohol.^52^ This effect was attributed to the higher vanadium load, which increases the energy barrier between the HOMO and LUMO orbitals and hampers the reducibility of the di-or tri-substituted heteropolyanions.^59^ As shown in Fig. 2, the same effect occurred herein.

In general, in the presence of the most active catalyst (*i.e.*, Na_4_PMo_11_VO_40_), benzaldehyde was quickly oxidized to benzoic acid and quickly esterified to ethyl benzoate; these two reactions were performed in a one-pot process, in the presence of aqueous H_2_O_2_ and ethyl alcohol solutions containing catalytic amounts of Na_4_PMo_11_VO_40_. Being the most active catalyst, it was selected to study the effects of the main reaction parameters in the next sections.

#### 3.2.2. Effect of the Na_4_PMo_11_VO_40_ catalyst load on benzaldehyde oxidative esterification reactions with H_2_O_2_

**Fig. 4:**
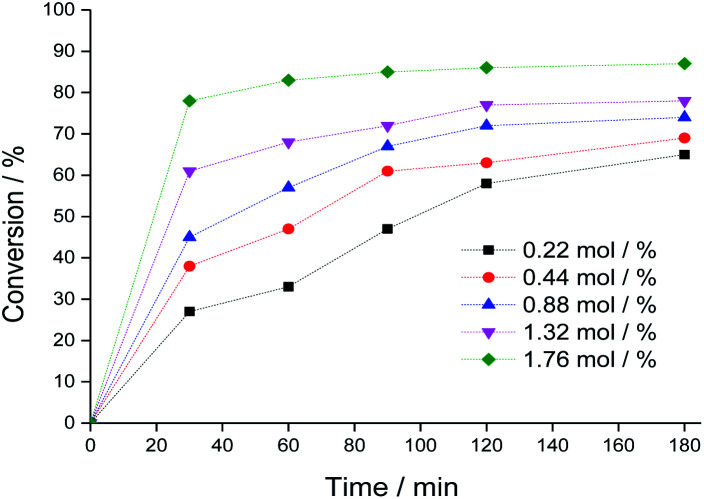
Impacts of Na_4_PMo_11_VO_40_ catalyst load on the kinetic curves of benzaldehyde oxidative esterification reactions with H_2_O_2_. Reaction conditions: benzaldehyde (2.75 mmol), H_2_O_2_ (8.25 mmol), toluene (internal stander), temperature (333 K), C_2_H_5_OH (10 mL).

**Fig. 5:**
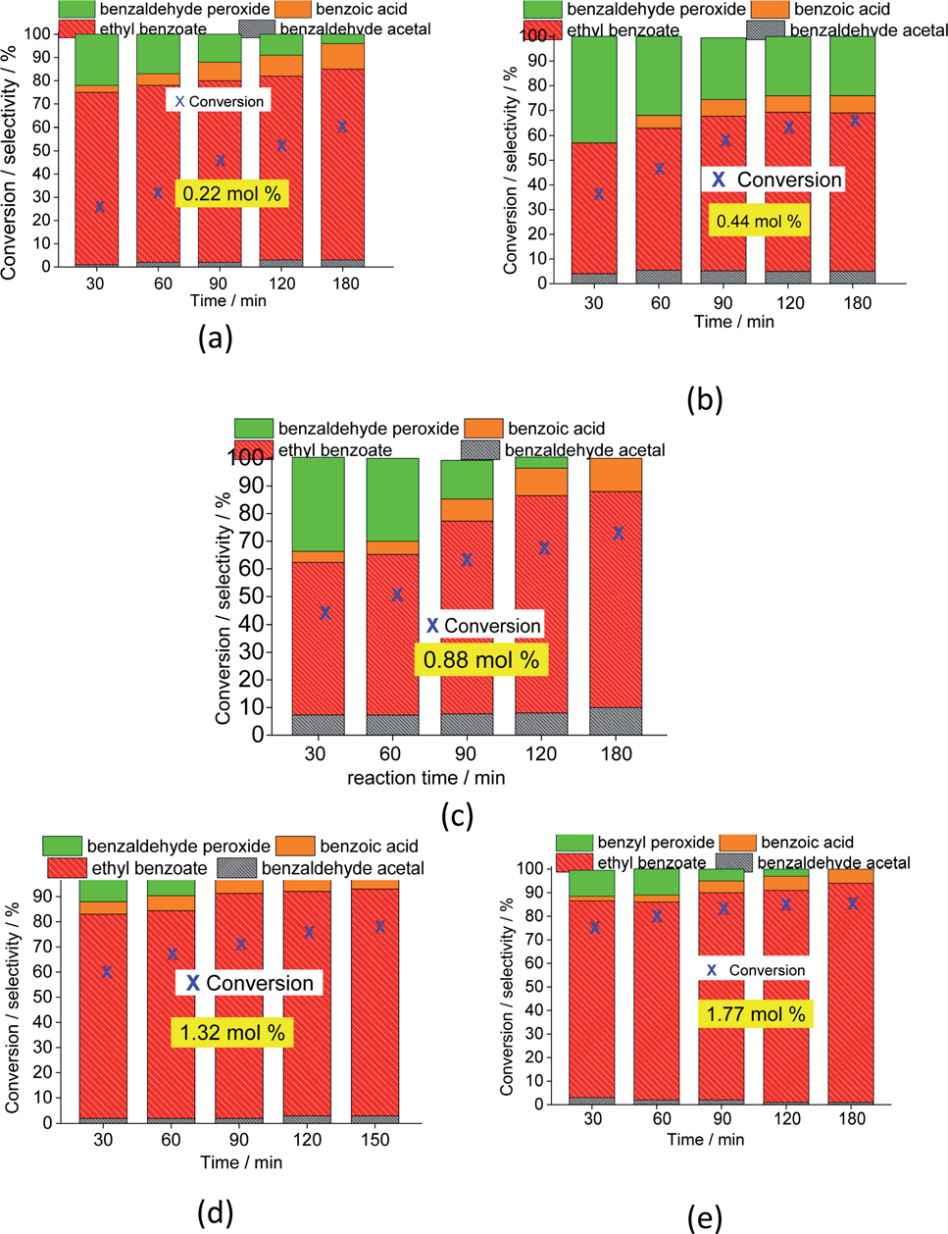
Impacts of the Na_4_PMo_11_VO_40_ catalyst load (a–e) on the conversion and product selectivity after 3 h oxidative esterification reactions of benzaldehyde with H_2_O_2_.

The catalytic activity of Na_4_PMo_11_VO_40_ was evaluated using different concentrations, and the main results are displayed in Fig. 4 (kinetic curves) and Fig. 5 (conversion and selectivity). An increase in catalyst load enhanced the initial rate of the reactions, and even though the kinetic curves had the same profile, the runs with a greater catalyst load achieved higher conversions after 3 h of reaction.

The selectivity of the reactions was also impacted by the catalyst load. It is possible to verify that an increase in catalyst load favored the conversion of benzaldehyde peroxide to oxidation products (*i.e.*, benzoic acid and their ethyl ester). Additionally, as the catalyst load increased, the benzoic acid was more efficiently esterified with ethyl alcohol (Fig. 5).

#### 3.2.3. Comparing the activity of the Na_4_PMo_11_VO_40_ catalyst and its precursors of synthesis in the oxidative esterification reactions of benzaldehyde with H_2_O_2_

**Fig. 6:**
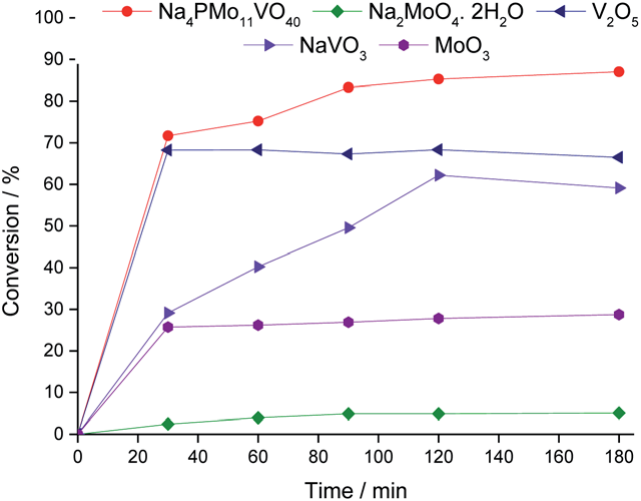
Kinetic curves of oxidative esterification reactions of benzaldehyde with H_2_O_2_ in C_2_H_5_OH solutions in the presence of the Na_4_PMo_11_VO_40_ catalyst and their synthesis precursors. Reaction conditions: benzaldehyde (2.75 mmol), H_2_O_2_ (8.25 mmol), catalyst (1.77 mol%), toluene (0.1 mL), C_2_H_5_OH (10.0 mL), 333 K.

Fig. 6 shows a comparison of the kinetic curve of the Na_4_PMo_11_VO_40_-catalyzed reaction with those obtained in the presence of its synthesis precursors. The initial rates of the reactions in the presence of vanadium catalysts were greater than those in the presence of the molybdenum catalysts. Moreover, higher conversions were obtained in these reactions. However, depending on the type of catalyst present in the reaction, the selectivity was strongly affected.

**Fig. 7:**
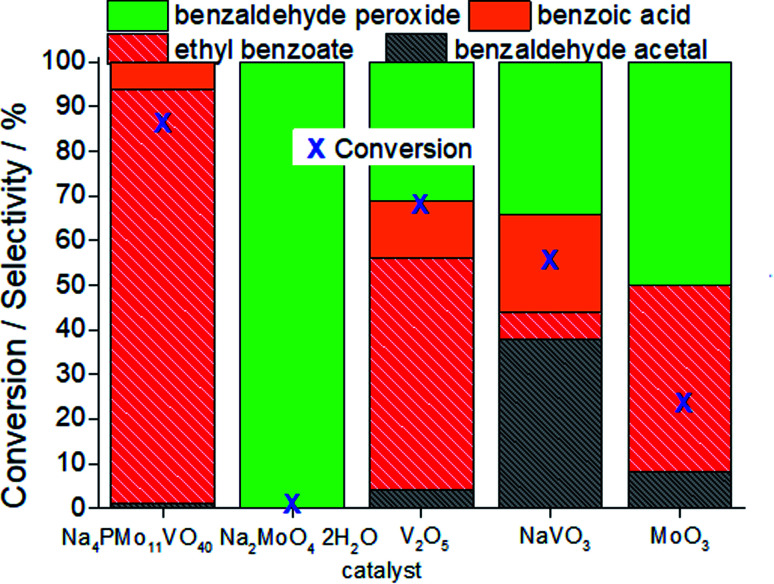
Conversion and selectivity after 3 h of oxidative esterification of benzaldehyde with H_2_O_2_ in C_2_H_5_OH solutions in the presence of the Na_4_PMo_11_VO_40_ catalyst or its synthesis precursors. Reaction conditions: benzaldehyde (2.75 mmol), H_2_O_2_ (8.25 mmol), catalyst (1.77 mol%), toluene (0.1 mL), C_2_H_5_OH (10.0 mL), 333 K.

In Fig. 7, it is possible to see that while sodium molybdate was almost inactive as a catalyst, the sodium vanadate-catalyzed reaction achieved a reasonable conversion (*ca.* 59%). Nonetheless, the condensation product (benzaldehyde acetal) was significantly formed. This catalyst was poorly effective in converting benzaldehyde peroxide to the acid or ester.

A comparison of the reactions in the presence of metal oxides allows us to conclude that the molybdenum and vanadium oxides were efficient to oxidatively esterify benzaldehyde, converting it to ethyl benzoate. However, the conversion of the V_2_O_5_-catalyzed reaction was much higher than that in the presence of the MoO_3_ catalyst.

Remarkably, when we compared the catalytic performance of the Na_4_PMo_11_VO_40_ salt to its precursors of synthesis, we can conclude that the vanadium atom plays a key role in the activity of the catalyst. In addition, this effect is even greater when vanadium is entrapped into the Keggin anion. Therefore, there is a synergism between the two species. Notably, as depicted in Fig. 1, if more than one vanadium atom was present in the Keggin anion, this effect was compromised.

#### 3.2.4. Discussion of the reaction mechanism involving the Na_4_PMoVO_40_ catalyst and hydrogen peroxide

When metals with a high oxidation number act in oxidation without hydrogen peroxide, such as Mo^6+^, W^6+^, Re^7+^, Ti^4+^, and V^5+^ cations, no stoichiometric oxidation of the substrate by the metal ion occurs. This effect was also verified herein; no oxidation product was formed in the solution containing only the Na_4_PMo_11_VO_40_ catalyst in the absence of hydrogen peroxide.

On the other hand, the literature describes that ethyl benzoate can also be obtained from benzaldehyde acetal in the presence of Lewis acid metal catalysts.^60^ Herein, we excluded this hypothesis by carrying out the reaction with the Na_4_PMoVO_40_ catalyst without hydrogen peroxide; although benzaldehyde acetal was selectively formed, no significant amount of ester was detected.

When used as catalysts in oxidation with hydrogen peroxide of alcohols or olefins, Keggin heteropolyacids can undergo a peroxidation step and generate peroxide intermediates, which are the most probable active species in these reactions.^61,62^

**Scheme 4:**
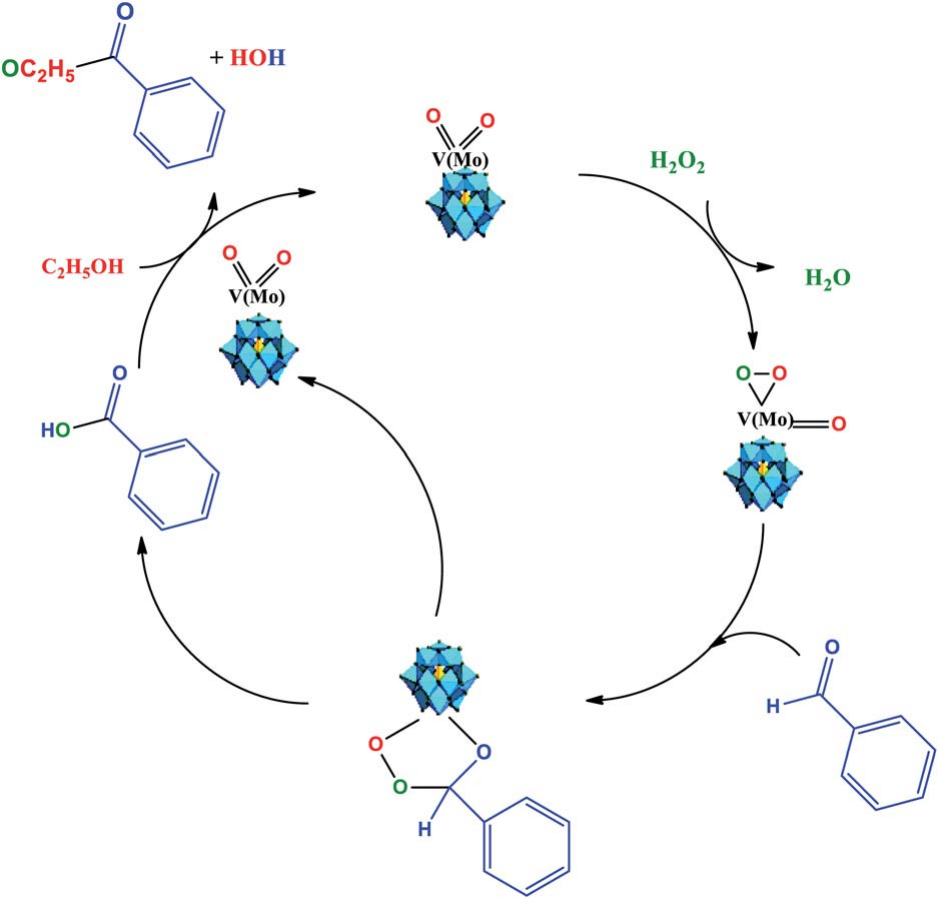
Reaction pathway of Na_4_PMo_11_VO_40_-catalyzed oxidative esterification of benzaldehyde with H_2_O_2_ in C_2_H_5_OH solution.

Recently, Patel *et al.* assessed the benzaldehyde oxidative esterification over Ni-exchanged supported phosphotungstic acid and proposed a reaction mechanism, which we suppose probably also operates herein.^61^ Therefore, as the basis of the literature and our experimental results, we propose that the oxidative esterification of the benzaldehyde can be described as depicted in Scheme 4.

We think that the addition of hydrogen peroxide to the solution containing the Na_4_PMoVO_40_ catalyst promotes its peroxidation, generating an intermediate that reacts with benzaldehyde and generates another intermediate, where the transfer of oxygen atom from the oxidant to the substrate is more favorable (Scheme 4).

This intermediate is decomposed, releasing the benzoic acid and the vanadium-doped phosphomolybdate catalyst. The catalyst can promote the interaction between ethyl alcohol and benzoic acid, probably through an intermediate (*i.e.*, omitted by simplification), in which nucleophilic attack on the carbonyl group of benzoic acid by the hydroxyl group of ethyl alcohol occurs, releasing the ester and water and regenerating the catalyst. It must be highlighted that both these steps take place *in situ*.^63^ Herein, it is probable that in the beginning, the formation of a peroxided active intermediate involves molybdenum or vanadium atoms.^52^ However, as previously reported, details of the mechanism of the oxidative esterification of aldehydes involving these POM catalysts are not clear yet.^60,61^

Two additional experiments were carried out to support this proposal. In the first experiment, the Na_4_PMoVO_40_ catalyst was evaluated in the oxidation reaction of benzaldehyde in acetonitrile. A conversion of 40% was achieved, with a selectivity of 60% toward benzoic acid. Secondly, the activity of the Na_4_PMoVO_40_ catalyst was evaluated in the esterification of benzoic acid with ethyl alcohol. A high ester selectivity (*ca.* 90%) toward ethyl benzoate was reached, at a rate of 45% conversion. These two tests confirm that this catalyst can efficiently promote both reactions.

#### 3.2.5. Effects of the molar ratio of the oxidant to the substrate

**Fig. 8:**
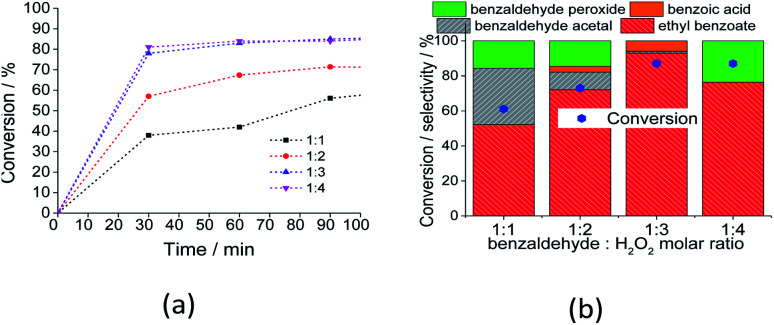
Impacts of the molar ratio of substrate to oxidant on the conversion (a) and selectivity (b) after 3 h of Na_4_PMo_11_VO_40_ catalyzed-oxidative esterification of benzaldehyde with H_2_O_2_ in C_2_H_5_OH solutions. Reaction conditions: benzaldehyde (2.75 mmol), catalyst (1.77 mol%), toluene (0.1 mL), C_2_H_5_OH (10.0 mL), 333 K.

The oxidant load played a key role in Na_4_PMoVO_40_-catalyzed benzaldehyde oxidation with H_2_O_2_. Fig. 8 presents the kinetic curves (Fig. 8a) and selectivity (Fig. 8b) obtained in reactions with molar ratios varying from 1 : 1 to 1 : 4.

An excess of oxidant increased both the initial rate and reaction conversion of the reactions. Similarly, the ester and acid selectivity were favored. This can be assigned to the reversible character of the esterification reaction, which is favored by higher amounts of reactants. However, at proportions greater than 1 : 3, the selectivity of the reaction was compromised; when a higher amount of water was present, the Lewis acidity of the catalyst was compromised (*i.e.*, V^5+^ and or Mo^6+^), and the conversion of benzaldehyde peroxide to acid and or ester became less favorable.

#### 3.2.6. Effect of alcohol on the Na_4_PMo_11_VO_40_-catalyzed oxidative esterification of benzaldehyde

**Fig. 9:**
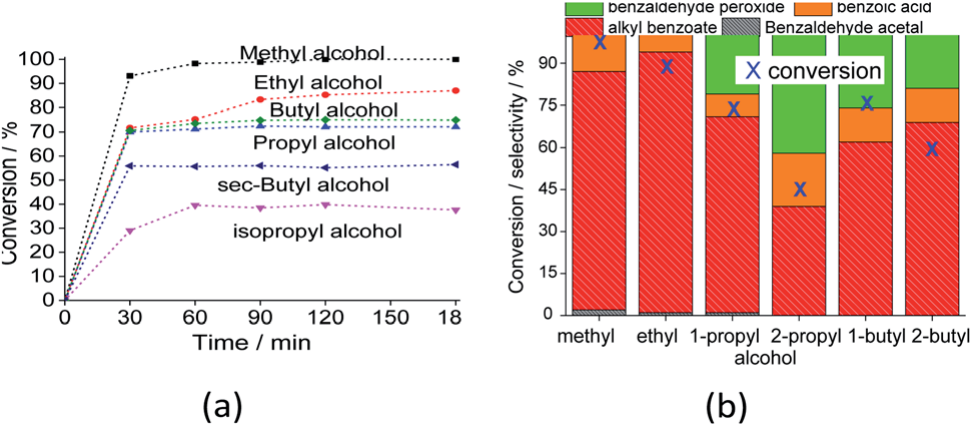
Impacts of alcohol on the conversion (a) and selectivity (b) (*i.e.*, after 3 h) of the Na_4_PMo_11_VO_40_-catalyzed-oxidative esterification reaction of benzaldehyde with H_2_O_2_. Reaction conditions: benzaldehyde (2.75 mmol), H_2_O_2_ (8.25 mmol), catalyst (1.77 mol%), toluene (0.1 mL), alcohol (10.0 mL), 333 K.

Alcohols with sterically hindered hydroxyl groups tend to be less reactive in esterification reactions. This may be a key aspect of both esterification and acetalization reactions. This effect can be noted in Fig. 9a, which shows that reactions with *sec*-propyl and *sec*-butyl alcohols had lower conversions.

The reaction selectivity was also affected by the increase in the size of the carbon chain and the steric hindrance on the hydroxyl group. Although benzoic ester was the major product in all the runs (Scheme 3), benzoic acid was also obtained.

**Scheme 5:**
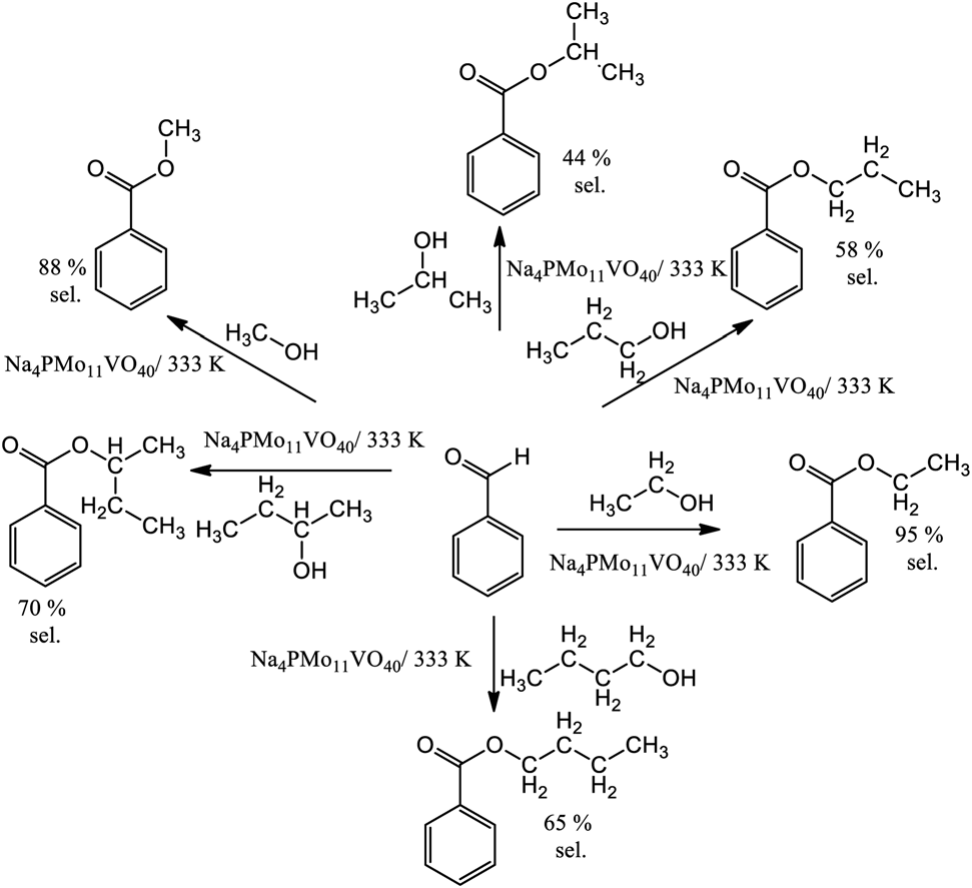
Ester selectivity of the Na_4_PMo_11_VO_40_-catalyzed oxidative esterification reactions of benzaldehyde with hydrogen peroxide in alcoholic medium. Reaction conditions: benzaldehyde (2.75 mmol); H_2_O_2_ (8.25 mmol), catalyst (1.77 mol%); reaction volume (10 mL).

In runs with less reactive alcohols (*i.e.*, with secondary hydroxyl groups or longer carbon chains), benzaldehyde peroxide, an intermediate product of oxidation, was the secondary product. The more hindered the hydroxyl group of the alcohol, the more difficult the attack on the carbonylic carbon of benzaldehyde, and consequently, the lower the ester selectivity (Scheme 5).

Likewise, when alcohols have a greater carbon chain size, the approach to the carbonylic carbon by the hydroxyl group is more difficult. The following trend was observed in terms of conversion of alcohols: CH_3_OH > C_2_H_5_OH > C_3_H_7_OH ≈ C_4_H_9_OH > *sec*-C_4_H_9_OH > *sec*-C_3_H_7_OH.

#### 3.2.7. Effect of temperature on the Na_4_PMo_11_VO_40_-catalyzed oxidative esterification of benzaldehyde

**Fig. 10:**
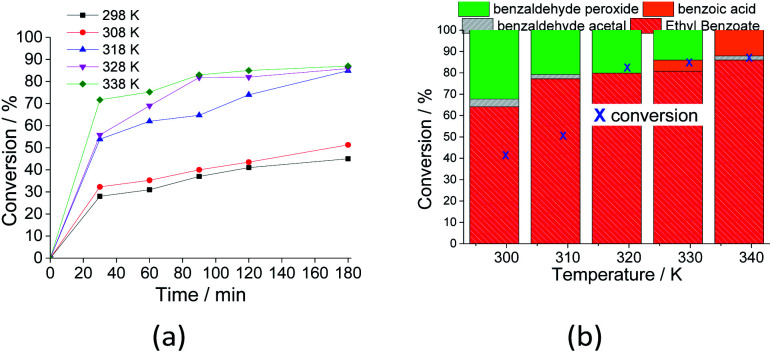
Effects of temperature on the kinetic curves (a) and conversion and selectivity (b) of the Na_4_PMo_11_VO_40_-catalyzed oxidative esterification of benzaldehyde by H_2_O_2_. Reaction conditions: benzaldehyde (2.75 mmol), H_2_O_2_ (8.25 mmol), (catalyst 1.77 mol%), temperature (variable); ethyl alcohol volume (10 mL).

The impacts of temperature on the conversion and selectivity were also investigated, and the main results are shown in Fig. 10. As expected, an increase in temperature means that a higher amount of energy can be provided to the reagent molecules; therefore, with a higher number of effective collisions, the initial reaction rates increased (Fig. 10a).

When the reaction was carried out at room temperature, 45% conversion was achieved, and the ester selectivity was 65%. Although not shown herein, in the absence of catalyst at this temperature, almost no conversion was detected, regardless of the excess of peroxide (*ca.* 1 : 4).

An increase in reaction temperature resulted in a higher conversion of benzaldehyde to ethyl benzoate as well as a drastic reduction in the amount of benzaldehyde peroxide at the end of the reaction, which was almost completely converted to the ester or benzoic acid (Fig. 10b). Under these reaction conditions, no significant difference was verified in the reactions carried out at 318 and 333 K.

## 4. Conclusions

A new route to oxidatively esterify benzaldehyde in a one-pot reaction with an environmentally friendly oxidant (*i.e.*, aqueous H_2_O_2_) using a vanadium-doped catalyst in alcoholic solutions was developed. Among the vanadium-doped salts assessed, Na_4_PMo_11_VO_40_ was the most active catalyst. The main aspects that drive the reaction selectivity were studied. Benzaldehyde was efficiently converted to esters (*ca.* 85–93% selectivity) in the presence of methyl and ethyl alcohols in 3 h of reaction at 333 K with a low oxidant excess (*ca.* 1 : 3). Benzaldehyde acetal and benzoic acid were the minor products. Benzaldehyde peroxide was also obtained, mainly when the metal catalyst was less efficient. Secondary alcohols and those with a chain carbon size greater than that of ethyl alcohol provided benzoate esters as the main products; benzoic acid was the minor product, and no traces of acetal were observed. Notably, it was demonstrated that vanadium-doping has a beneficial effect only when one vanadium atom is used. The activity of the Na_4_PMo_11_VO_40_ catalyst was compared to its synthesis precursors, revealing that the Mo and V atoms have a synergic effect which efficiently promotes the oxidative esterification of benzaldehyde.

## Conflicts of interest

There are no conflicts to declare.

## Supplementary Material

RA-011-D1RA06718D-s001
