# Supplementary material for: Na4PMo11VO40-catalyzed one-pot oxidative esterification of benzaldehyde with hydrogen peroxide
Source: RSC Adv. 2021 Oct 28;11(55):34979–87. doi: 10.1039/d1ra06718d (PMC9042929; doi:10.1039/d1ra06718d)
Supplement: RA-011-D1RA06718D-s001 [file RA-011-D1RA06718D-s001.pdf]

Electronic Supplementary Material (ESI) for RSC Advances.
This journal is © The Royal Society of Chemistry 2021

$Na_4PMo_{11}VO_{40}$-catalyzed one-pot oxidative esterification of benzaldehyde with hydrogen peroxide

CASTELO BANDANE VILANCULO*[1] MÁRCIO JOSÉ DA SILVA[2]

[1]*Chemistry Department, Pedagogic University of Mozambique, FCNM, Campus of Lhanguene, Av. de Moçambique, Km 1, Maputo, zip-code: 4040*

[2]*Chemistry Department, Federal University of Viçosa, Viçosa, Minas Gerais, Brasil. zip-code: 36590-000*

Supplemental material

List of Figures

Figure 1SM. FT-IR spectra of Sodium phosphomolybdate salts unsubstituted and Vanadium substituted.

Fig. 2SM Powder XRD of Vanadium (mono, di-, or tri)-substituted Sodium phosphomolybdate salts

Fig. 3SM. Potentiometric titration curves with n-butylamine of undoped and Vanadium-doped sodium phosphomolybdate salts

Figure 4SM. Scanning electronic microscopy images of $Na_3PMo_{12}O_{40}$ (a), $Na_4PMo_{11}V_1O_{40}$ (b), $Na_5PMo_{10}V_2O_{40}$, and $Na_6PMo_9V_3O_{40}$ (d)

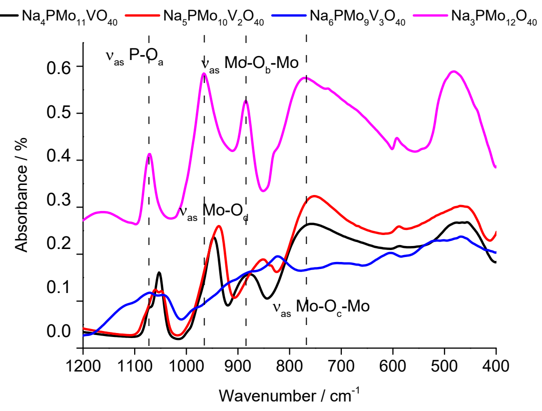

Figure 1SM. FT-IR/ATR spectra of undoped and doped-Vanadium phosphomolybdic salts

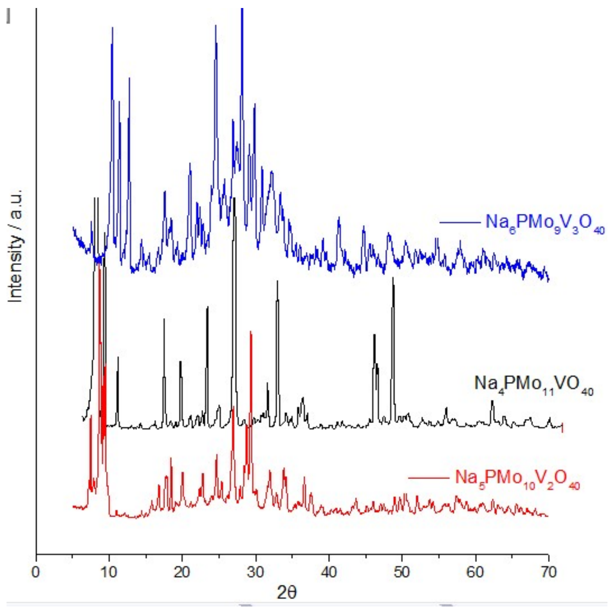

Fig. 2SM Powder XRD of Vanadium (mono, di-, or tri)-substituted Sodium phosphomolybdate salts

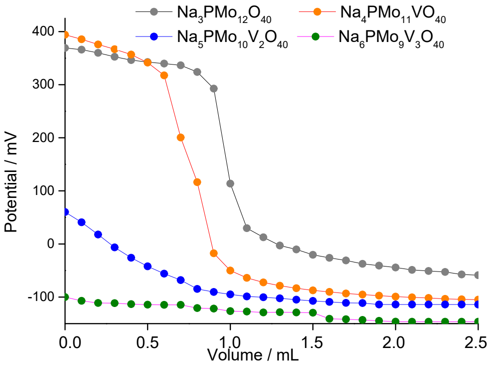

Fig. 3SM. Potentiometric titration curves with n-butylamine of Undoped and Vanadium-doped sodium phosphomolybdate salts

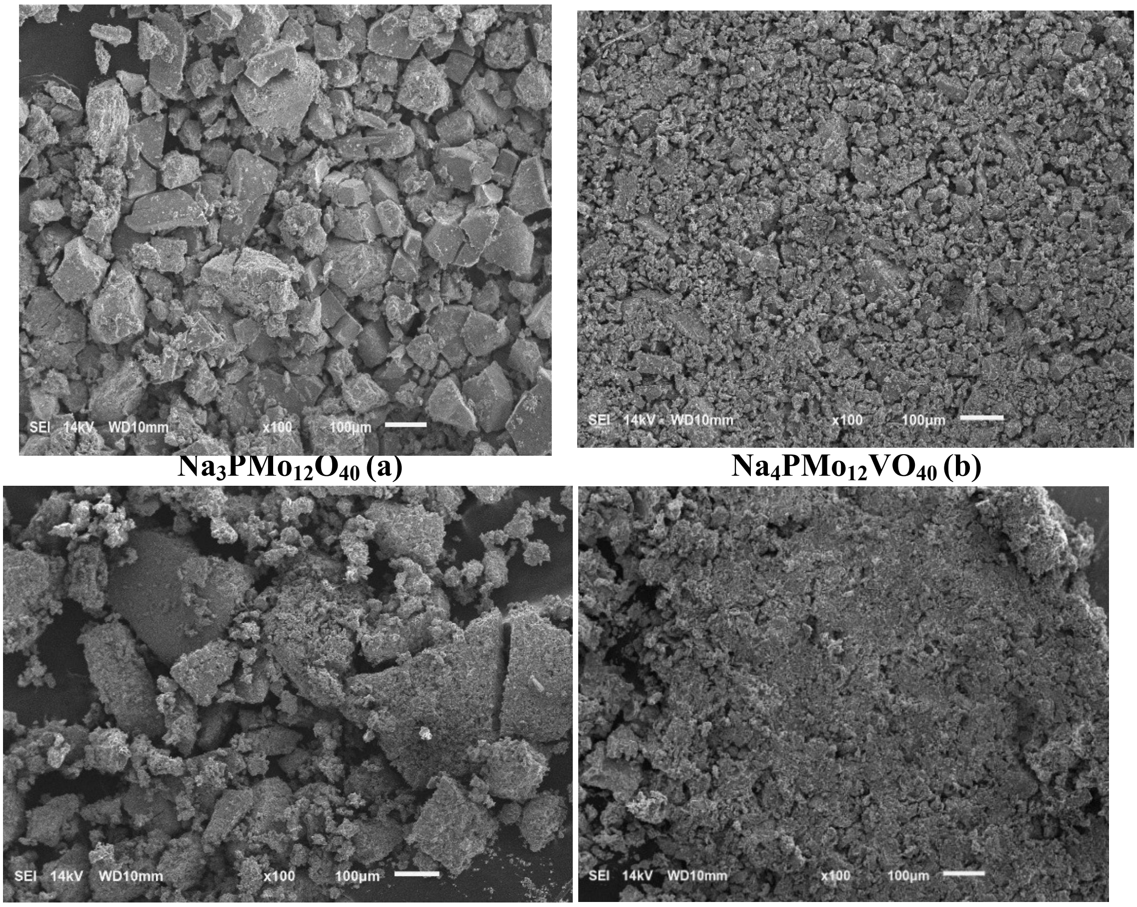

$Na_3PMo_{12}O_{40}$ (a) **$Na_4PMo_{12}VO_{40}$ (b)**

$Na_5PMo_{10}V_2O_{40}$ (c) $Na_6PMo_9V_3O_{40}$ (d)

Figure 4SM. Scanning electronic microscopy images of $Na_3PMo_{12}O_{40}$ (a), $Na_4PMo_{11}V_1O_{40}$ (b), $Na_5PMo_{10}V_2O_{40}$, and $Na_6PMo_9V_3O_{40}$ (d)
